# Lipoprotein(a) and its linear association with all-cause and cardiovascular mortality in patients with acute coronary syndrome

**DOI:** 10.3389/fendo.2025.1580298

**Published:** 2025-05-27

**Authors:** Ke Qin, Tingyuan Zhang

**Affiliations:** Department of Critical Care Medicine, Henan Provincial People’s Hospital, Zhengzhou, China

**Keywords:** lipoprotein(a), acute coronary syndrome, all-cause mortality, cardiovascular mortality, restricted cubic spline

## Abstract

**Objective:**

This study aimed to investigate the linear association between lipoprotein(a) [Lp(a)] levels and all-cause and cardiovascular mortality in patients with acute coronary syndrome (ACS).

**Methods:**

This retrospective cohort study included 578 patients with ACS who were hospitalized at Henan Provincial People’s Hospital between January 2020 and January 2024. Patients were categorized into two groups: lower Lp(a) group (≤ 300 mg/L) and higher Lp(a) group (> 300 mg/L). Kaplan-Meier survival analysis, Cox regression models, subgroup and sensitivity analyses were used to evaluate the association between Lp(a) and all-cause and cardiovascular mortality. Restricted cubic spline (RCS) analysis was conducted to explore nonlinear associations.

**Results:**

During a median follow-up of 27.5 months, a total of 124 all-cause deaths occurred (21.5%), of which 79 cases (13.7%) were classified as cardiovascular deaths. Compared to the lower Lp(a) group, the higher Lp(a) group exhibited a significantly increased risk of all-cause and cardiovascular mortality across all models. In the fully adjusted model (Model 3), the hazard ratio (HR) for all-cause mortality was 1.719 (95% confidence interval [CI]: 1.197–2.470, P = 0.003), while the HR for cardiovascular mortality was 2.505 (95% CI: 1.529-4.102, P < 0.001). In an additional analysis using a 500 mg/L cut-off, patients with Lp(a) > 500 mg/L had a significantly higher risk of cardiovascular mortality (HR = 2.209, P = 0.001), while the association with all-cause mortality (P = 0.284) was not statistically significant in the fully adjusted model. When Lp(a) was analyzed as a continuous variable, each 90 mg/L increase in Lp(a) was associated with a 5% higher risk of all-cause mortality (HR = 1.052, 95% CI: 1.003-1.104, P = 0.038), and each 45 mg/L increase was associated with a 5% higher risk of cardiovascular mortality (HR = 1.054, 95% CI: 1.026-1.084, P < 0.001). For log10-transformed Lp(a), the HR was 1.954 (95% CI: 1.252-3.050, P = 0.003) for all-cause mortality and 3.913 (95% CI: 2.108-7.265, P < 0.001) for cardiovascular mortality. Similarly, for standardized Lp(a) (Z-score), the HR was 1.178 (95% CI: 1.009-1.375, P = 0.038) for all-cause mortality and 1.408 (95% CI: 1.179-1.681, P < 0.001) for cardiovascular mortality. Most subgroup analyses showed that elevated Lp(a) levels were significantly associated with an increased risk of all-cause and cardiovascular mortality (P < 0.05). Sensitivity analyses confirmed the robustness of the findings, with significant associations persisting after excluding patients with early mortality or without stent implantation. Kaplan-Meier analysis showed that both all-cause and cardiovascular survival rates were significantly lower in the high Lp(a) group compared to the low Lp(a) group (P < 0.001 for both). RCS analyses revealed a linear positive association between Lp(a) levels and both all-cause and cardiovascular mortality.

**Conclusions:**

Higher Lp(a) levels were independently and linearly associated with an increased risk of all-cause and cardiovascular mortality in ACS patients.

## 1 Introduction

Acute coronary syndrome (ACS) is a group of acute cardiovascular events caused by the rupture or erosion of atherosclerotic plaques in the coronary arteries, representing a major contributor to cardiovascular-related mortality and disease burden worldwide (1). Epidemiological data indicate that millions of ACS patients are hospitalized annually, and the long-term mortality rate remains high, particularly among high-risk individuals with heart failure, chronic kidney disease (CKD), or diabetes (2–4). Despite significant improvements in ACS prognosis due to percutaneous coronary intervention (PCI), pharmacological treatment (such as antiplatelet agents, lipid-lowering drugs, and β-blockers), a substantial proportion of patients still experience major adverse cardiovascular events (MACE) or death during follow-up (5–7). Current evidence has confirmed that certain biomarkers, such as those related to inflammation, can independently predict the risk of cardiovascular events in patients with ACS (8–10). However, even after controlling for these known risk factors, residual cardiovascular risk remains, suggesting the need to identify additional risk indicators. Therefore, identifying other residual cardiovascular risk factors that can accurately predict long-term mortality risk in ACS patients is crucial for optimizing individualized risk assessment and developing more effective treatment strategies.

Lipoprotein(a) [Lp(a)] is a cholesterol-rich low-density lipoprotein (LDL)-like particle that is structurally unique due to its apolipoprotein B-100 (ApoB-100), which is covalently linked via disulfide bonds to apolipoprotein(a) [Apo(a)] (11). Lp(a) is primarily synthesized and secreted by the liver into the bloodstream, with serum levels varying widely among individuals, mainly determined by genetic factors (LPA gene polymorphisms) (12). Unlike other lipid parameters, Lp(a) levels are relatively unaffected by lifestyle factors or commonly used lipid-lowering medications, such as statins (13). Lp(a) has significant pro-atherogenic, pro-inflammatory, and pro-thrombotic properties, which may accelerate the development of coronary artery disease (CAD) and its complications through multiple mechanisms (14). Given these mechanisms, elevated Lp(a) levels in ACS patients may significantly impact their prognosis and increase the risk of all-cause mortality.

Lp(a) has been extensively studied in relation to cardiovascular disease (CVD) onset and progression, with substantial evidence supporting its role as an independent risk factor (15–17). However, the specific relationship between Lp(a) and all-cause mortality remains controversial, particularly in the ACS patient population. Some studies have reported a significant positive correlation between elevated Lp(a) levels and higher all-cause mortality, while others have not observed such an association (18–21). These discrepancies may be attributed to heterogeneity in study populations, differences in follow-up durations, variations in Lp(a) measurement methods, and inconsistencies in statistical approaches. Additionally, most prior studies have relied on categorical analysis of Lp(a) (e.g., using thresholds of 30 mg/dL or 50 mg/dL) rather than exploring its linear association with all-cause mortality. Therefore, one of the primary objectives of this study was to use continuous variable analysis to clarify the dose-response relationship between Lp(a) and all-cause and cardiovascular mortality and to investigate its clinical significance in ACS patients.

Based on this background, we hypothesize that Lp(a) levels are linearly and positively associated with all-cause and cardiovascular mortality in ACS patients. The objectives of this study are as follows: (1) to evaluate the association between Lp(a) levels and all-cause and cardiovascular mortality and determine whether Lp(a) serves as an independent predictor; (2) to validate the robustness of this association by using different continuous variable analysis strategies for Lp(a) (raw values, log-transformed values, and standardized values); (3) to examine this relationship across different clinical subgroups (such as age, sex, hypertension, diabetes, CKD); and (4) to explore the potential nonlinearity of this association to determine whether the relationship follows a linear pattern. The findings of this study will contribute to a better understanding of the role of Lp(a) in predicting all-cause and cardiovascular mortality risk in ACS patients and provide valuable insights for optimizing individualized treatment and follow-up strategies.

## 2 Methods

### 2.1 Study population

This retrospective cohort study was conducted at Henan Provincial People’s Hospital, including patients enrolled between January 2020 and January 2024. A total of 578 participants were included and categorized into two groups based on their Lp(a) levels: the lower Lp(a) group (n = 350) and the higher Lp(a) group (n = 228). Patients were included in the study if they met the following criteria: (a) Aged ≥ 18 years at the time of enrollment; (2) Underwent serum Lp(a) measurement at the hospital; (3) Had complete baseline clinical data, including demographics, laboratory results, and medical history; (4) Had follow-up data available for mortality outcomes. Patients were excluded from the study based on the following criteria: (1) Missing key data, including Lp(a) levels or mortality status; (2) History of malignancy, autoimmune diseases, or severe infections that could affect Lp(a) levels; (3) Severe hepatic or renal dysfunction at baseline; (4) Loss to follow-up or withdrawal from the study. This study was approved by the Ethics Committee of Henan Provincial People’s Hospital and was conducted in accordance with the Declaration of Helsinki. Informed consent was obtained from all participants or their legal representatives before data collection.

### 2.2 Measurement of lipoprotein(a)

Serum Lp(a) levels were measured using an immunoturbidimetric assay, a widely used method for the quantitative determination of Lp(a), performed on an automated biochemical analyzer following the manufacturer’s standardized protocol. The reference range for Lp(a) was 0–300 mg/L. Based on this threshold, participants were categorized into two groups: those with Lp(a) ≤ 300 mg/L were classified as the lower Lp(a) group, while those with Lp(a) > 300 mg/L were assigned to the higher Lp(a) group. In addition, an analysis was conducted using a cut-off value of Lp(a) = 500 mg/L, according to which patients were further divided into two groups: Lp(a) ≤ 500 mg/L and Lp(a) > 500 mg/L.

In statistical analyses, Lp(a) was treated as both a categorical and a continuous variable. For continuous analysis, three approaches were used: (1) raw Lp(a) values in mg/L, (2) log10-transformed Lp(a) values to normalize skewed distributions, and (3) standardized Lp(a) values (Z-scores) to facilitate effect size comparisons across different models.

### 2.3 Outcome assessment

The primary outcome of this study was all-cause mortality, defined as death from any cause occurring during the follow-up period. In addition, cardiovascular mortality was analyzed as a secondary outcome, defined as death resulting from cardiovascular causes, including ACS, sudden cardiac death, heart failure, stroke, and other fatal cardiovascular events. All patients were followed from the time of hospital discharge until death or the end of the study on January 2025. Mortality data were obtained from hospital records and verified through follow-up assessments, including phone interviews and electronic medical records.

### 2.4 Collection and definition of covariates

Baseline demographic and clinical characteristics were collected from medical records, including age, gender, types of ACS (including ST-segment elevation myocardial infarction (STEMI), non-ST-segment elevation myocardial infarction, and unstable angina pectoris), smoking status, and body mass index (BMI). Hypertension was defined as a self-reported history of hypertension, systolic blood pressure (SBP) ≥ 140 mmHg, diastolic blood pressure (DBP) ≥ 90 mmHg, or the use of antihypertensive medications (22). Diabetes was defined as a self-reported history of diabetes, fasting glucose ≥ 7.0 mmol/L, glycated hemoglobin (HbA1c) ≥ 6.5%, or the use of insulin or oral hypoglycemic drugs (23). Hyperlipidemia was defined as a self-reported history of hyperlipidemia, total cholesterol (TC) ≥ 5.2 mmol/L, low-density lipoprotein cholesterol (LDL-C) ≥ 3.4 mmol/L, triglycerides ≥ 1.7 mmol/L, or the use of lipid-lowering agents (24). CKD was defined as an estimated glomerular filtration rate (eGFR) < 60 mL/min/1.73m^2^ or a history of CKD (25).

Coronary severity was assessed using the Gensini score, which quantifies the extent of coronary artery stenosis, and the number of diseased vessels. Left ventricular ejection fraction (LVEF) was measured via echocardiography to assess cardiac function. Stent implantation status was recorded to identify patients who had undergone PCI. Laboratory parameters were obtained from fasting blood samples, including TC, LDL-C, high-density lipoprotein cholesterol (HDL-C), triglycerides, apolipoprotein A1 (ApoA1), apolipoprotein B (ApoB), eGFR, uric acid, fibrinogen, albumin, and fasting glucose. Medication use was documented, including antiplatelet agents (aspirin, ticagrelor), lipid-lowering drugs (statins), antihypertensive drugs [beta-blockers, angiotensin-converting enzyme inhibitors (ACEI), angiotensin receptor blockers (ARB), calcium channel blockers (CCB)], diuretics (furosemide, spironolactone), and antidiabetic medications (insulin, oral hypoglycemic drugs).

### 2.5 Statistical methods

Baseline characteristics were compared between the lower and higher Lp(a) groups using the chi-square test for categorical variables and the independent t-test for normally distributed continuous variables or the Wilcoxon rank-sum test for non-normally distributed continuous variables. The normality of continuous variables was assessed using the Shapiro-Wilk test. Kaplan-Meier survival analysis was conducted to compare cumulative all-cause and cardiovascular mortality between the two Lp(a) groups, and differences in survival curves were assessed using the log-rank test. Cox proportional hazards regression models were used to assess the association between Lp(a) and all-cause and cardiovascular mortality, with hazard ratios (HRs) and 95% confidence intervals (CIs) reported. Three models were constructed: Model 1 adjusted for age and gender; Model 2 further adjusted for smoking, hypertension, diabetes, and CKD; and Model 3 further adjusted for ApoA1, ApoB, albumin, uric acid, eGFR, fibrinogen, LVEF, and Gensini score. Subgroup analyses were performed to evaluate potential effect modifications by age, gender, hypertension, diabetes, CKD, and STEMI. Sensitivity analyses were conducted by excluding patients who died within two years of follow-up or had a follow-up duration of less than two years or those without stent implantation. Restricted cubic spline (RCS) models were used to explore potential nonlinear associations between Lp(a) and all-cause and cardiovascular mortality. All statistical analyses were performed using SPSS version 26.0 and R version 4.3.4, with a two-tailed P-value < 0.05 considered statistically significant.

## 3 Results

### 3.1 Clinical characteristics

**Figure 1:**
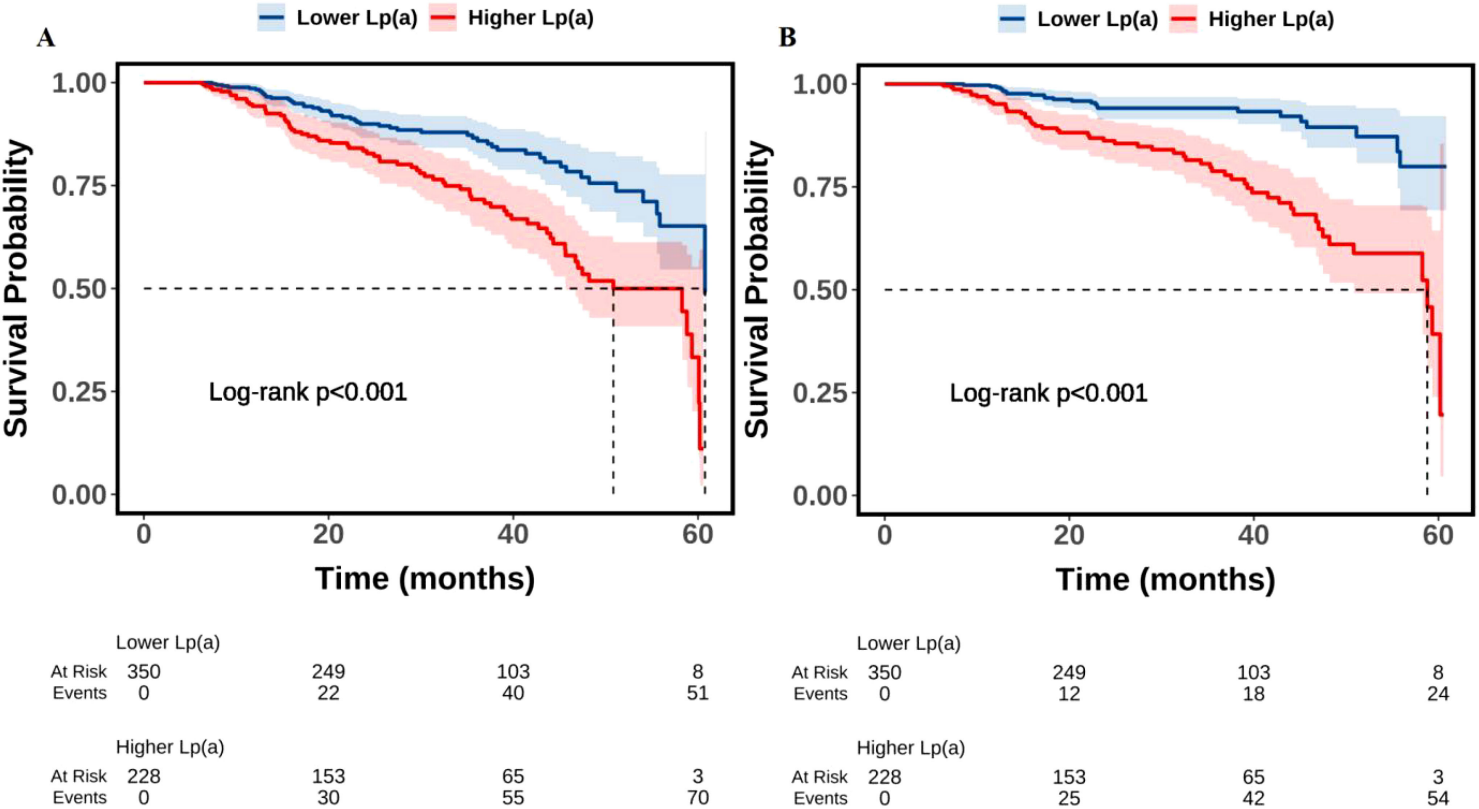
Kaplan-Meier survival analysis of the association between Lp(a) and all-cause **(A)** and cardiovascular **(B)** mortality. Lp(a), lipoprotein(a).

As shown in **Table 1**, the total population consisted of 578 individuals with a mean age of 65.0 years, including 459 males (79.4%). Compared to the lower Lp(a) group (≤ 300 mg/L), the higher Lp(a) group (> 300 mg/L) was older (P < 0.001), exhibited a higher prevalence of CKD (P = 0.001), showed a lower eGFR (P < 0.001), presented with a higher Gensini score (P < 0.001) and a greater number of diseased vessels (P < 0.001), demonstrated a lower LVEF (P = 0.042), recorded higher SBP (P = 0.017), displayed elevated TC (P = 0.007), LDL-C (P < 0.001), and ApoB levels (P = 0.002), showed increased fibrinogen levels (P < 0.001), and experienced a higher all-cause and cardiovascular mortality rate (P < 0.001). During a median follow-up of 27.5 months, a total of 124 all-cause deaths occurred (21.5%), of which 79 cases (13.7%) were classified as cardiovascular deaths. The number of events per variable (EPV) in the fully adjusted Cox model was approximately 9, indicating an acceptable level of model stability. **Figure 1** presented the results of Kaplan-Meier survival analysis evaluating the association between serum Lp(a) levels and all-cause mortality (**Figure 1A**) as well as cardiovascular mortality (**Figure 1B**). **Figure 1A** showed that the all-cause survival rate during the follow-up period was significantly lower in the high Lp(a) group compared to the low Lp(a) group, with a statistically significant difference between the survival curves (Log-rank test, P < 0.001). **Figure 1B** further indicated that cardiovascular survival was also markedly lower in the high Lp(a) group, showing a continuously declining trend throughout the follow-up period, with a significant difference compared to the low Lp(a) group (Log-rank P < 0.001).

**Table 1:** Baseline characteristics grouped by lipoprotein(a).

	Total population	Lower lipoprotein(a) (≤ 300 mg/L)	Higher lipoprotein(a) (> 300 mg/L)	P value
N	578	350	228	
Age, years	65.00 (54.00, 75.00)	63.00 (53.00, 73.00)	69.00 (56.00, 77.00)	< 0.001
Gender, n (%)				0.665
Male	459 (79.40%)	280 (80.00%)	179 (78.50%)	
Female	119 (20.60%)	70 (20.00%)	49 (21.50%)	
STEMI, n (%)	290 (50.20%)	180 (51.40%)	110 (48.20%)	
Smoking, n (%)				0.205
Yes	250 (43.30%)	144 (41.10%)	106 (46.50%)	
No	328 (56.70%)	206 (58.90%)	122 (53.50%)	
Hypertension, n (%)				0.071
Yes	417 (72.10%)	243 (69.40%)	174 (76.30%)	
No	161 (27.90%)	107 (30.60%)	54 (23.70%)	
Hyperlipidemia, n (%)				0.367
Yes	251 (43.50%)	147 (42.00%)	104 (45.80%)	
No	326 (56.50%)	203 (58.00%)	123 (54.20%)	
Diabetes, n (%)				0.803
Yes	222 (38.40%)	133 (38.00%)	89 (39.00%)	
No	356 (61.60%)	217 (62.00%)	139 (61.00%)	
CKD, n (%)				0.001
Yes	116 (20.10%)	55 (15.70%)	61 (26.80%)	
No	462 (79.90%)	295 (84.30%)	167 (73.20%)	
Gensini score	80.00 (46.00, 110.00)	72.00 (42.00, 98.00)	90.00 (56.00, 120.00)	< 0.001
Number of diseased vessels	3.00 (2.00, 3.00)	3.00 (2.00, 3.00)	3.00 (2.00, 3.00)	< 0.001
Stent implantation, n (%)	503 (87.00%)	301 (86.00%)	202 (88.60%)	0.364
LVEF, %	57.00 (46.00, 66.00)	58.00 (48.00, 67.00)	55.00 (44.30, 65.50)	0.042
SBP, mmHg	129.00 (114.00, 144.00)	126.00 (113.00, 142.00)	133.00 (113.50, 145.50)	0.017
DBP, mmHg	78.00 (68.00, 86.00)	78.00 (69.75, 85.00)	76.00 (66.50, 88.00)	0.657
BMI, kg/m^2^	24.56 (22.49, 27.05)	24.59 (22.49, 27.06)	24.30 (22.18, 26.72)	0.140
Triglyceride, mmol/L	1.40 (1.02, 2.05)	1.39 (1.07, 1.98)	1.41 (0.98, 2.15)	0.937
Total cholesterol, mmol/L	4.44 (3.80, 5.26)	4.35 (3.70, 5.11)	4.57 (3.91, 5.47)	0.007
LDL-C, mmol/L	2.68 (2.19, 3.35)	2.61 (2.16, 3.19)	2.84 (2.34, 3.60)	< 0.001
HDL-C, mmol/L	1.13 (0.97, 1.30)	1.14 (0.97, 1.31)	1.11 (0.95, 1.29)	0.215
Apolipoprotein A1, g/L	1.06 ± 0.22	1.07 ± 0.22	1.05 ± 0.22	0.217
Apolipoprotein B, g/L	0.86 (0.71, 1.02)	0.84 (0.69, 0.98)	0.90 (0.74, 1.07)	0.002
Uric acid, umol/L	346.00 (281.00, 436.00)	336.50 (282.75, 418.25)	358.00 (273.00, 466.50)	0.162
eGFR, ml/min/1.73m^2^	93.32 (65.55, 120.33)	95.83 (73.70, 124.61)	84.02 (57.25, 112.06)	< 0.001
Albumin, g/L	37.77 ± 4.01	37.97 ± 3.78	37.47 ± 4.32	0.155
Fasting glucose, mmol/L	6.42 (5.47, 8.28)	6.42 (5.44, 8.08)	6.37 (5.47, 8.30)	0.974
Fibrinogen, g/L	3.80 (3.10, 4.53)	3.62 (3.00, 4.22)	4.16 (3.31, 4.80)	< 0.001
Medication, n (%)				
Aspirin	538 (93.10%)	327 (93.40%)	211 (92.50%)	0.682
Ticagrelor	407 (70.40%)	254 (72.60%)	153 (67.10%)	0.159
Statin	553 (95.70%)	333 (95.10%)	220 (96.50%)	0.436
Beta blocker	476 (82.40%)	290 (82.90%)	186 (81.60%)	0.694
ACEI/ARB	315 (54.50%)	183 (52.30%)	132 (57.90%)	0.186
Calcium channel blocker	89 (15.40%)	48 (13.70%)	41 (18.00%)	0.165
Furosemide	227 (39.30%)	131 (37.40%)	96 (42.10%)	0.260
Spironolactone	230 (39.80%)	134 (38.30%)	96 (42.10%)	0.359
Insulin	95 (16.40%)	56 (16.00%)	39 (17.10%)	0.726
Oral hypoglycemic drugs	136 (23.50%)	84 (24.00%)	52 (22.80%)	0.741
All-cause mortality				< 0.001
Yes	124 (21.50%)	52 (14.90%)	72 (31.60%)	
No	454 (78.50%)	298 (85.10%)	156 (68.40%)	
Cardiovascular mortality				< 0.001
Yes	79 (13.70%)	24 (6.90%)	55 (24.10%)	
No	499 (86.30%)	326 (93.10%)	173 (75.90%)	

STEMI, ST-segment elevation myocardial infarction; CKD, chronic kidney disease; LVEF, left ventricular ejection fraction; SBP, systolic blood pressure; DBP, diastolic blood pressure; BMI, body mass index; LDL-C, low-density lipoprotein cholesterol; HDL-C, high-density lipoprotein cholesterol; eGFR, estimated glomerular filtration rate; ACEI, angiotensin converting enzyme inhibitors; ARB, angiotensin receptor blocker.

### 3.2 Multivariable association between lipoprotein(a) and all-cause mortality

As shown in **Table 2**, compared to the lower Lp(a) group (≤ 300 mg/L), the higher Lp(a) group (> 300 mg/L) exhibited a significantly increased risk of all-cause and cardiovascular mortality across all models. For all-cause mortality, the HRs were 1.862 (95% CI: 1.299-2.669, P = 0.001) in Model 1 (adjusted for age and gender), 1.848 (95% CI: 1.290–2.648, P = 0.001) in Model 2 (further adjusted for smoking, hypertension, diabetes, and CKD), and 1.719 (95% CI: 1.197-2.470, P = 0.003) in Model 3 (fully adjusted for clinical and laboratory variables). Correspondingly, for cardiovascular mortality, the HRs were 3.065 (95% CI: 1.895-4.960, P < 0.001), 3.027 (95% CI: 1.871-4.897, P < 0.001), and 2.505 (95% CI: 1.529-4.102, P < 0.001) across the three models, respectively.

**Table 2:** Multivariable association between lipoprotein(a) and all-cause and cardiovascular mortality.

	Model 1		Model 2		Model 3	
	HR (95% CI)	P value	HR (95% CI)	P value	HR (95% CI)	P value
All-cause mortality						
Lower Lp(a) (≤ 300 mg/L)	Ref		Ref		Ref	
Higher Lp(a) (> 300 mg/L)	1.862 (1.299, 2.669)	0.001	1.848 (1.290, 2.648)	0.001	1.719 (1.197, 2.470)	0.003
Lp(a) ≤ 500 mg/L	Ref		Ref		Ref	
Lp(a) > 500 mg/L	1.602 (1.094, 2.344)	0.015	1.647 (1.124, 2.412)	0.010	1.256 (0.828, 1.904)	0.284
Lp(a)	1.001 (1.000, 1.001)	0.001	1.001 (1.000, 1.001)	0.005	1.001 (1.000, 1.001)	0.038
Log_10_Lp(a)	2.297 (1.463, 3.608)	< 0.001	2.118 (1.357, 3.304)	0.001	1.954 (1.252, 3.050)	0.003
Standardized Lp(a)	1.274 (1.103, 1.470)	0.001	1.229 (1.065, 1.418)	0.005	1.178 (1.009, 1.375)	0.038
Lp(a)^a^	1.078 (1.031, 1.127)	0.001	1.066 (1.020, 1.114)	0.005	1.052 (1.003, 1.104)	0.038
Cardiovascular mortality						
Lower Lp(a) (≤ 300 mg/L)	Ref		Ref		Ref	
Higher Lp(a) (> 300 mg/L)	3.065 (1.895, 4.960)	< 0.001	3.027 (1.871, 4.897)	< 0.001	2.505 (1.529, 4.102)	< 0.001
Lp(a) ≤ 500 mg/L	Ref		Ref		Ref	
Lp(a) > 500 mg/L	2.611 (1.662, 4.100)	< 0.001	2.785 (1.770, 4.382)	< 0.001	2.209 (1.384, 3.526)	0.001
Lp(a)	1.001 (1.001, 1.002)	< 0.001	1.001 (1.001, 1.002)	< 0.001	1.001 (1.001, 1.002)	< 0.001
Log_10_Lp(a)	4.815 (2.582, 8.979)	< 0.001	4.421 (2.367, 8.256)	< 0.001	3.913 (2.108, 7.265)	< 0.001
Standardized Lp(a)	1.538 (1.307, 1.810)	< 0.001	1.483 (1.260, 1.747)	< 0.001	1.408 (1.179, 1.681)	< 0.001
Lp(a)^b^	1.069 (1.042, 1.096)	< 0.001	1.063 (1.036, 1.090)	< 0.001	1.054 (1.026, 1.084)	< 0.001

^a^: Lp(a) was divided by 90, representing the HR per 90 mg/L increase in Lp(a), which corresponds to approximately a 5% increase in all-cause mortality risk. ^b^: Lp(a) was divided by 45, representing the HR per 45 mg/L increase in Lp(a), which corresponds to approximately a 5% increase in cardiovascular mortality risk.

Model 1: Adjusted for age and gender.

Model 2: Adjusted for age, gender, smoking, hypertension, diabetes, and chronic kidney disease.

Model 3: Adjusted for Model 2 + apolipoprotein A1, apolipoprotein B, albumin, uric acid, estimated glomerular filtration rate, fibrinogen, left ventricular ejection fraction, and Gensini score.

Lp(a), lipoprotein(a); HR, hazard ratio; CI, confidence interval.

To further explore the prognostic value of more widely recognized clinical thresholds, an additional analysis was conducted using 500 mg/L as the cut-off point. Compared to patients with Lp(a) ≤ 500 mg/L, those with Lp(a) > 500 mg/L exhibited a significantly higher risk of all-cause mortality in Models 1 and 2, with HRs of 1.602 (95% CI: 1.094-2.344, P = 0.015) and 1.647 (95% CI: 1.124-2.412, P = 0.010), respectively. However, in the fully adjusted Model 3, the association was attenuated and did not reach statistical significance (HR = 1.256, 95% CI: 0.828-1.904, P = 0.284). In contrast, for cardiovascular mortality, the elevated risk associated with Lp(a) > 500 mg/L remained statistically significant across all three models: HRs were 2.611 (95% CI: 1.662-4.100, P < 0.001) in Model 1, 2.785 (95% CI: 1.770-4.382, P < 0.001) in Model 2, and 2.209 (95% CI: 1.384-3.526, P = 0.001) in Model 3.

When Lp(a) was analyzed as a continuous variable, elevated levels were significantly associated with increased risks of both all-cause and cardiovascular mortality across all models. For all-cause mortality, the HRs in Model 1 were 1.001 (95% CI: 1.000-1.001, P = 0.001) for Lp(a), 2.297 (95% CI: 1.463-3.608, P < 0.001) for log_10_Lp(a), and 1.274 (95% CI: 1.103-1.470, P = 0.001) for standardized Lp(a); in Model 2, the HRs were 1.001 (95% CI: 1.000-1.001, P = 0.005), 2.118 (95% CI: 1.357-3.304, P = 0.001), and 1.229 (95% CI: 1.065-1.418, P = 0.005), respectively; and in Model 3, the HRs were 1.001 (95% CI: 1.000-1.001, P = 0.038), 1.954 (95% CI: 1.252-3.050, P = 0.003), and 1.178 (95% CI: 1.009-1.375, P = 0.038). Similarly, for cardiovascular mortality, the HRs in Model 1 were 1.001 (95% CI: 1.001-1.002, P < 0.001), 4.815 (95% CI: 2.582-8.979, P < 0.001), and 1.538 (95% CI: 1.307-1.810, P < 0.001); in Model 2, they were 1.001 (95% CI: 1.001-1.002, P < 0.001), 4.421 (95% CI: 2.367-8.256, P < 0.001), and 1.483 (95% CI: 1.260-1.747, P < 0.001); and in Model 3, the HRs were 1.001 (95% CI: 1.001-1.002, P < 0.001), 3.913 (95% CI: 2.108-7.265, P < 0.001), and 1.408 (95% CI: 1.179-1.681, P < 0.001), respectively.

When Lp(a)/90 and Lp(a)/45 were used as standardized variables in the analysis, the results showed that every 90 mg/L increase in Lp(a) was associated with approximately a 5% higher risk of all-cause mortality (HR = 1.052, 95% CI: 1.003-1.104, P = 0.038), and every 45 mg/L increase in Lp(a) was associated with approximately a 5% higher risk of cardiovascular mortality (HR = 1.054, 95% CI: 1.026-1.084, P < 0.001).

### 3.3 Subgroup analysis

Subgroup analyses of **Table 3** demonstrated that elevated Lp(a) levels were significantly associated with increased all-cause mortality in patients aged ≥ 60 years (higher Lp(a) vs lower Lp(a): HR = 1.785, P = 0.003; log_10_Lp(a): HR = 1.917, P = 0.006; standardized Lp(a): HR = 1.223, P = 0.009), males (HR = 1.892, P = 0.005; standardized Lp(a): HR = 1.240, P = 0.043), patients with hypertension (HR = 1.502, P = 0.045; log_10_Lp(a): HR = 1.860, P = 0.011), without hypertension (HR = 4.001, P = 0.009; Lp(a): HR = 1.002, P = 0.003; log_10_Lp(a): HR = 6.546, P = 0.002; standardized Lp(a): HR = 1.671, P = 0.003), without diabetes (HR = 1.967, P = 0.009; Lp(a): HR = 1.001, P = 0.014; log_10_Lp(a): HR = 2.422, P = 0.012; standardized Lp(a): HR = 1.318, P = 0.014), and without CKD (log_10_Lp(a): HR = 2.509, P = 0.006), as well as in STEMI patients (HR = 2.408, P = 0.003; log_10_Lp(a): HR = 2.896, P = 0.005).

**Table 3:** Subgroup analysis of the association between lipoprotein(a) and all-cause and cardiovascular mortality.

	Higher Lp(a) vs Lower Lp(a)		Lp(a)		Log_10_Lp(a)		Standardized Lp(a)	
	HR (95% CI)	P value	HR (95% CI)	P value	HR (95% CI)	P value	HR (95% CI)	P value
All-cause mortality								
Age								
< 60 years	1.523 (0.151, 15.364)	0.721	1.003 (0.999, 1.007)	0.212	4.126 (0.146, 116.331)	0.405	2.139 (0.648, 7.059)	0.212
≥ 60 years	1.785 (1.224, 2.603)	0.003	1.001 (1.000, 1.001)	0.009	1.917 (1.208, 3.042)	0.006	1.223 (1.052, 1.423)	0.009
Gender								
Male	1.892 (1.214, 2.949)	0.005	1.001 (1.000, 1.001)	0.043	1.463 (0.772, 2.771)	0.244	1.240 (1.007, 1.526)	0.043
Female	1.363 (0.586, 3.172)	0.472	1.000 (0.999, 1.001)	0.402	2.097 (0.854, 5.152)	0.106	1.134 (0.845, 1.523)	0.402
Hypertension								
Yes	1.502 (1.008, 2.237)	0.045	1.000 (1.000, 1.001)	0.501	1.860 (1.155, 2.995)	0.011	1.066 (0.884, 1.286)	0.501
No	4.001 (1.407, 11.371)	0.009	1.002 (1.001, 1.003)	0.003	6.546 (1.974, 21.702)	0.002	1.671 (1.189, 2.348)	0.003
Diabetes								
Yes	1.134 (0.617, 2.084)	0.685	1.000 (0.999, 1.001)	0.857	1.211 (0.597, 2.457)	0.596	0.977 (0.755, 1.264)	0.857
No	1.967 (1.187, 3.260)	0.009	1.001 (1.000, 1.002)	0.014	2.422 (1.213, 4.839)	0.012	1.318 (1.058, 1.640)	0.014
Chronic kidney disease								
Yes	1.553 (0.853, 2.826)	0.150	1.001 (1.000, 1.001)	0.240	1.761 (0.858, 3.616)	0.123	1.145 (0.913, 1.435)	0.240
No	1.282 (0.717, 2.292)	0.403	1.000 (0.999, 1.001)	0.509	2.509 (1.302, 4.833)	0.006	1.097 (0.833, 1.447)	0.509
STEMI								
Yes	2.408 (1.358, 4.268)	0.003	1.001 (1.000, 1.002)	0.112	2.896 (1.378, 6.088)	0.005	1.216 (0.955, 1.547)	0.112
No	1.030 (0.575, 1.844)	0.922	1.000 (0.999, 1.001)	0.487	1.169 (0.590, 2.316)	0.654	1.099 (0.842, 1.435)	0.487
Cardiovascular mortality								
Age								
< 60 years	0.223 (0.004, 13.784)	0.476	1.003 (0.996, 1.010)	0.452	4.810 (0.024, 970.165)	0.562	2.229 (0.276, 18.007)	0.452
≥ 60 years	2.559 (1.521, 4.305)	< 0.001	1.001 (1.001, 1.002)	< 0.001	3.749 (1.979, 7.101)	< 0.001	1.415 (1.182, 1.694)	< 0.001
Gender								
Male	2.446 (1.297, 4.615)	0.006	1.001 (1.000, 1.002)	0.008	2.767 (1.152, 6.643)	0.023	1.421 (1.097, 1.840)	0.008
Female	2.204 (0.746, 6.513)	0.153	1.001 (1.000, 1.002)	0.017	5.346 (1.900, 15.041)	0.001	1.377 (1.060, 1.790)	0.017
Hypertension								
Yes	2.126 (1.264, 3.574)	0.004	1.001 (1.000, 1.002)	0.006	3.299 (1.709, 6.369)	< 0.001	1.323 (1.083, 1.615)	0.006
No	5.318 (1.001, 28.237)	0.050	1.003 (1.001, 1.005)	0.001	6.886 (1.143, 41.493)	0.035	2.437 (1.463, 4.060)	0.001
Diabetes								
Yes	1.485 (0.691, 3.191)	0.311	1.000 (0.999, 1.001)	0.516	1.659 (0.654, 4.208)	0.287	1.109 (0.812, 1.516)	0.516
No	3.252 (1.532, 6.904)	0.002	1.002 (1.001, 1.003)	< 0.001	6.965 (2.439, 19.891)	< 0.001	1.692 (1.327, 2.157)	< 0.001
Chronic kidney disease								
Yes	2.070 (1.087, 3.940)	0.027	1.001 (1.000, 1.002)	0.036	2.296 (1.030, 5.121)	0.042	1.266 (1.015, 1.578)	0.036
No	3.829 (1.764, 8.313)	0.001	1.002 (1.001, 1.003)	< 0.001	9.599 (3.372, 27.327)	< 0.001	1.913 (1.473, 2.484)	< 0.001
STEMI								
Yes	3.529 (1.739, 7.163)	< 0.001	1.001 (1.000, 1.002)	0.020	4.464 (1.823, 10.927)	0.001	1.331 (1.046, 1.694)	0.020
No	1.739 (0.771, 3.921)	0.183	1.001 (1.000, 1.002)	0.013	2.268 (0.804, 6.402)	0.122	1.390 (1.071, 1.803)	0.013

STEMI, ST-segment elevation myocardial infarction; Lp(a), lipoprotein(a); HR, hazard ratio; CI, confidence interval.

For cardiovascular mortality, significant associations were observed in patients aged ≥ 60 years (HR = 2.559, P < 0.001; Lp(a): HR = 1.001, P < 0.001; log_10_Lp(a): HR = 3.749, P < 0.001; standardized Lp(a): HR = 1.415, P < 0.001), males (HR = 2.446, P = 0.006; Lp(a): HR = 1.001, P = 0.008; log_10_Lp(a): HR = 2.767, P = 0.023; standardized Lp(a): HR = 1.421, P = 0.008), and females (Lp(a): HR = 1.001, P = 0.017; log_10_Lp(a): HR = 5.346, P = 0.001; standardized Lp(a): HR = 1.377, P = 0.017). Similar associations were found in patients with hypertension (HR = 2.126, P = 0.004; Lp(a): HR = 1.001, P = 0.006; log_10_Lp(a): HR = 3.299, P < 0.001; standardized Lp(a): HR = 1.323, P = 0.006), without hypertension (Lp(a): HR = 1.003, P = 0.001; log_10_Lp(a): HR = 6.886, P = 0.035; standardized Lp(a): HR = 2.437, P = 0.001), without diabetes (HR = 3.252, P = 0.002; Lp(a): HR = 1.002, P < 0.001; log_10_Lp(a): HR = 6.965, P < 0.001; standardized Lp(a): HR = 1.692, P < 0.001), with CKD (HR = 2.070, P = 0.027; Lp(a): HR = 1.001, P = 0.036; log_10_Lp(a): HR = 2.296, P = 0.042; standardized Lp(a): HR = 1.266, P = 0.036), and without CKD (HR = 3.829, P = 0.001; Lp(a): HR = 1.002, P < 0.001; log_10_Lp(a): HR = 9.599, P < 0.001; standardized Lp(a): HR = 1.913, P < 0.001). In terms of ACS type, both STEMI (HR = 3.529, P < 0.001; Lp(a): HR = 1.001, P = 0.020; log_10_Lp(a): HR = 4.464, P = 0.001; standardized Lp(a): HR = 1.331, P = 0.020) and non-STEMI patients (Lp(a): HR = 1.001, P = 0.013; standardized Lp(a): HR = 1.390, P = 0.013) demonstrated significant associations.

### 3.4 Sensitivity analysis

In the sensitivity analysis excluding patients with mortality or follow-up duration less than two years (**Table 4**), the associations between elevated Lp(a) levels and mortality remained robust in the fully adjusted model (Model 3). For all-cause mortality, the higher Lp(a) group showed a significantly increased risk compared to the lower Lp(a) group (HR = 2.424, 95% CI: 1.420-4.138, P = 0.001). Continuous variable analyses also demonstrated significant associations: Lp(a) (HR = 1.001, P = 0.002), log10-transformed Lp(a) (HR = 3.646, P < 0.001), and standardized Lp(a) (HR = 1.389, P = 0.002). For cardiovascular mortality, the higher Lp(a) group had a markedly increased risk compared to the lower group (HR = 5.083, 95% CI: 2.199-11.752, P < 0.001). Similarly, significant associations were observed for Lp(a) as a continuous variable (HR = 1.002, P < 0.001), log10-transformed Lp(a) (HR = 13.424, P < 0.001), and standardized Lp(a) (HR = 1.855, P < 0.001).

**Table 4:** Sensitivity analysis: exclusion of patients with mortality or follow-up duration less than two years.

	Model 1		Model 2		Model 3	
	HR (95% CI)	P value	HR (95% CI)	P value	HR (95% CI)	P value
All-cause mortality						
Lower Lp(a)	Ref		Ref		Ref	
Higher Lp(a)	2.458 (1.441, 4.193)	0.001	2.510 (1.471, 4.282)	0.001	2.424 (1.420, 4.138)	0.001
Lp(a)	1.001 (1.000, 1.002)	0.001	1.001 (1.000, 1.002)	0.003	1.001 (1.000, 1.002)	0.002
Log_10_Lp(a)	3.892 (1.904, 7.955)	< 0.001	3.684 (1.814, 7.482)	< 0.001	3.646 (1.784, 7.452)	< 0.001
Standardized Lp(a)	1.404 (1.139, 1.730)	0.001	1.370 (1.114, 1.685)	0.003	1.389 (1.126, 1.715)	0.002
Cardiovascular mortality						
Lower Lp(a)	Ref		Ref		Ref	
Higher Lp(a)	5.298 (2.304, 12.179)	< 0.001	5.472 (2.372, 12.623)	< 0.001	5.083 (2.199, 11.752)	< 0.001
Lp(a)	1.002 (1.001, 1.003)	< 0.001	1.002 (1.001, 1.003)	< 0.001	1.002 (1.001, 1.003)	< 0.001
Log_10_Lp(a)	13.480 (4.298, 42.273)	< 0.001	13.191 (4.304, 40.427)	< 0.001	13.424 (4.240, 42.497)	< 0.001
Standardized Lp(a)	1.832 (1.410, 2.379)	< 0.001	1.791 (1.381, 2.322)	< 0.001	1.855 (1.428, 2.408)	< 0.001

Model 1: Adjusted for age and gender.

Model 2: Adjusted for age, gender, smoking, hypertension, diabetes, and chronic kidney disease.

Model 3: Adjusted for Model 2 + apolipoprotein A1, apolipoprotein B, albumin, uric acid, estimated glomerular filtration rate, fibrinogen, left ventricular ejection fraction, and Gensini score.

Lp(a), lipoprotein(a); HR, hazard ratio; CI, confidence interval.

In the sensitivity analysis excluding patients without stent implantation (**Table 5**), for all-cause mortality, the higher Lp(a) group showed a significantly elevated risk compared to the lower group (HR = 1.922, 95% CI: 1.281-2.885, P = 0.002). Significant associations were also observed for Lp(a) as a continuous variable (HR = 1.001, P = 0.006), log10-transformed Lp(a) (HR = 2.267, P = 0.002), and standardized Lp(a) (HR = 1.268, P = 0.006). For cardiovascular mortality, the higher Lp(a) group had a significantly increased risk (HR = 2.924, 95% CI: 1.690-5.061, P < 0.001), with consistent associations across continuous forms: Lp(a) (HR = 1.001, P < 0.001), log_10_Lp(a) (HR = 4.015, P < 0.001), and standardized Lp(a) (HR = 1.505, P < 0.001).

**Table 5:** Sensitivity analysis: excluding patients without stent implantation.

	Model 1		Model 2		Model 3	
	HR (95% CI)	P value	HR (95% CI)	P value	HR (95% CI)	P value
All-cause mortality						
Lower Lp(a)	Ref		Ref		Ref	
Higher Lp(a)	1.970 (1.312, 2.960)	0.001	1.941 (1.292, 2.914)	0.001	1.922 (1.281, 2.885)	0.002
Lp(a)	1.001 (1.000, 1.001)	0.002	1.001 (1.000, 1.001)	0.004	1.001 (1.000, 1.001)	0.006
Log_10_Lp(a)	2.474 (1.480, 4.136)	0.001	2.310 (1.376, 3.878)	0.002	2.267 (1.362, 3.774)	0.002
Standardized Lp(a)	1.304 (1.107, 1.536)	0.002	1.281 (1.083, 1.514)	0.004	1.268 (1.072, 1.500)	0.006
Cardiovascular mortality						
Lower Lp(a)	Ref		Ref		Ref	
Higher Lp(a)	3.203 (1.852, 5.540)	< 0.001	3.092 (1.788, 5.346)	< 0.001	2.924 (1.690, 5.061)	< 0.001
Lp(a)	1.002 (1.001, 1.002)	< 0.001	1.001 (1.001, 1.002)	< 0.001	1.001 (1.001, 1.002)	< 0.001
Log_10_Lp(a)	4.855 (2.393, 9.850)	< 0.001	4.436 (2.161, 9.105)	< 0.001	4.015 (1.979, 8.148)	< 0.001
Standardized Lp(a)	1.557 (1.292, 1.877)	< 0.001	1.528 (1.257, 1.857)	< 0.001	1.505 (1.226, 1.848)	< 0.001

Model 1: Adjusted for age and gender.

Model 2: Adjusted for age, gender, smoking, hypertension, diabetes, and chronic kidney disease.

Model 3: Adjusted for Model 2 + apolipoprotein A1, apolipoprotein B, albumin, uric acid, estimated glomerular filtration rate, fibrinogen, left ventricular ejection fraction, and Gensini score.

Lp(a), lipoprotein(a); HR, hazard ratio; CI, confidence interval.

### 3.5 Restricted cubic spline analysis

**Figure 2:**
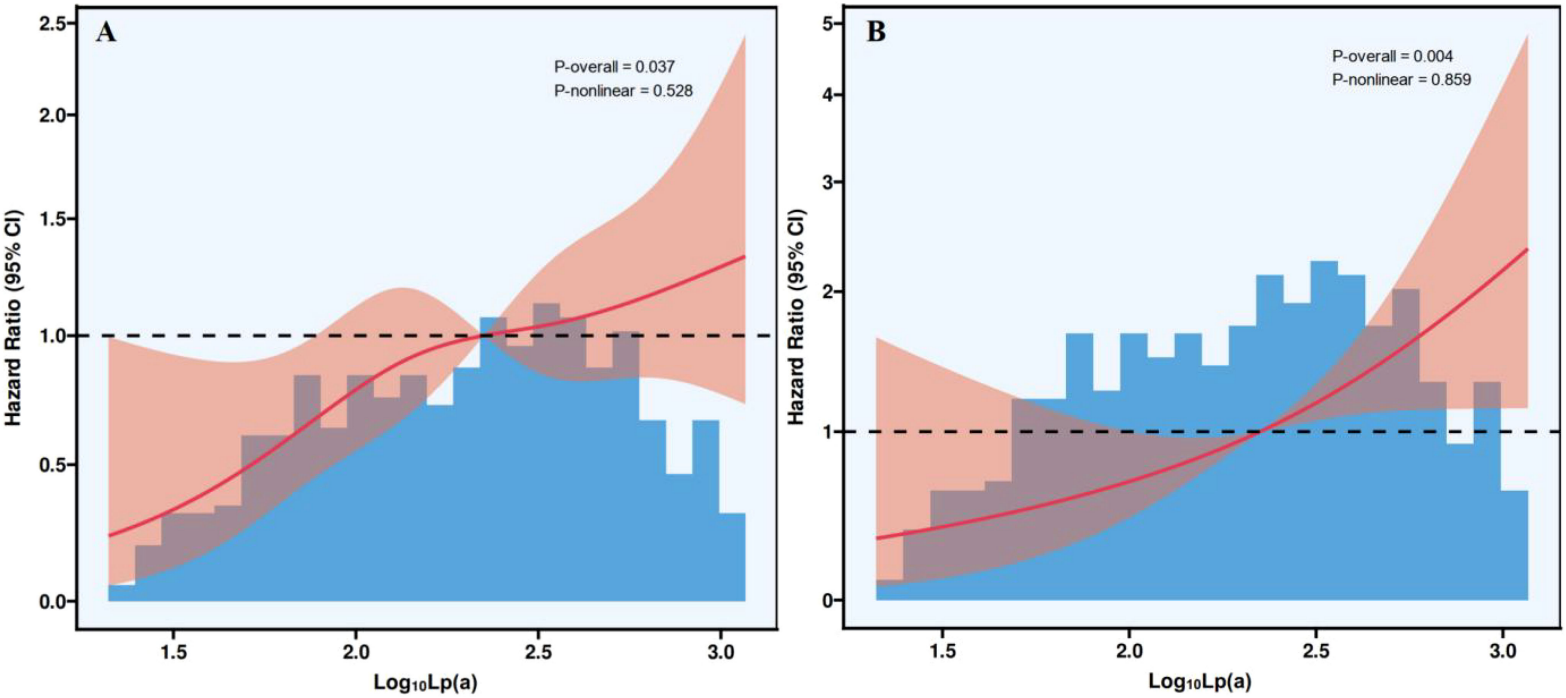
Restricted cubic spline plots of the association between Lp(a) and all-cause **(A)** and cardiovascular **(B)** mortality. Lp(a), lipoprotein(a); CI, confidence interval.

**Figure 2** presented the RCS analysis of the association between Lp(a) and all-cause and cardiovascular mortality. In the fully adjusted model, which included age, gender, smoking, hypertension, diabetes, CKD, ApoA1, ApoB, albumin, uric acid, eGFR, fibrinogen, LVEF, and Gensini score, Lp(a) remained linearly and positively associated with all-cause (**Figure 2A**) and cardiovascular (**Figure 2B**) mortality (P-nonlinear = 0.528 and 0.859).

## 4 Discussion

This study systematically analyzed the linear association between Lp(a) levels and all-cause and cardiovascular mortality in patients with ACS. The results indicate that elevated Lp(a) levels are an independent risk factor for increased all-cause and cardiovascular mortality. Compared to the Lp(a) ≤ 300 mg/L group, the Lp(a) > 300 mg/L group exhibited a significantly higher risk of all-cause and cardiovascular mortality. In the additional analysis using the 500 mg/L threshold, Lp(a) > 500 mg/L was also significantly associated with increased cardiovascular mortality, although the association with all-cause mortality was not statistically significant after full adjustment. When Lp(a) was analyzed as a continuous variable, each 90 mg/L increase in Lp(a) was associated with a 5% higher risk of all-cause mortality, and each 45 mg/L increase was associated with a 5% higher risk of cardiovascular mortality. Additionally, analyses based on log10-transformed Lp(a) and standardized Z-score further supported this trend. Subgroup analyses revealed that elevated Lp(a) levels were significantly associated with increased all-cause mortality in patients aged ≥ 60 years, males, those with or without hypertension, those without diabetes or CKD, and in STEMI patients. For cardiovascular mortality, significant associations were observed across a broader range of subgroups, including both males and females, patients aged ≥ 60 years, individuals with or without hypertension, diabetes, or CKD, as well as both STEMI and non-STEMI populations. Sensitivity analyses confirmed the robustness of these findings. Notably, RCS analysis did not reveal a nonlinear trend, further supporting the stable linear relationship between Lp(a) levels and all-cause and cardiovascular mortality. Overall, this study underscores the potential clinical value of Lp(a) as a prognostic biomarker in ACS patients and provides critical evidence for improving cardiovascular risk management.

Lp(a) is a genetically determined lipoprotein, and its association with CVD has been confirmed in multiple international studies (26–29). Large-scale prospective studies, such as the UK Biobank and the Copenhagen General Population Study, have demonstrated that elevated Lp(a) levels are closely related to an increased risk of all-cause mortality and cardiovascular events (30–32). Besides, based on a prospective study of the general Danish population, Langsted et al. analyzed 69,764 individuals with Lp(a) measurements and found that Lp(a) levels >93 mg/dL (199 nmol/L, 96th-100th percentiles) were associated with an increased risk of cardiovascular and all-cause mortality compared to Lp(a) < 10 mg/dL (18 nmol/L, 1st-50th percentiles), with a median survival reduction of 1.2 years (33). For every 50 mg/dL (105 nmol/L) increase in Lp(a), the observational HR for cardiovascular mortality was 1.16, while the genetic risk was 1.23 (based on LPA KIV-2) and 0.98 (based on LPA rs10455872). This suggests that elevated Lp(a) levels increase mortality risk primarily through a lower number of LPA KIV-2 repeats rather than cholesterol content. Furthermore, in a systematic review and meta-analysis conducted by Amiri et al., which included 75 studies with a total of 957,253 participants, the association between Lp(a) and mortality risk was investigated (19). The results showed that, in both the general population and CVD patients, the highest Lp(a) level group had all-cause mortality risk ratios of 1.09 and 1.18, respectively. Elevated Lp(a) levels were also associated with an increased risk of CVD-related mortality, a trend that was evident in the general population, CVD patients, and individuals with diabetes. For every 50 mg/dL increase in Lp(a), the risk of CVD-related mortality increased by 31% in the general population and by 15% in CVD patients. These findings support the European Society of Cardiology (ESC) and European Atherosclerosis Society (EAS) guidelines, which recommend that all adults measure Lp(a) at least once to assess mortality risk. Additionally, Wohlfahrt et al. examined 851 patients with acute myocardial infarction (AMI) and found a U-shaped relationship between Lp(a) levels and all-cause mortality (16). Compared to those with Lp(a) levels of 10–30 nmol/L, individuals with Lp(a) < 7 nmol/L and those with Lp(a) ≥ 125 nmol/L had an increased risk of mortality. Moreover, both low and high Lp(a) levels were associated with an increased risk of recurrent cardiovascular events, and this association was partially attenuated by heart failure-related factors. Another study not only identified an association between Lp(a) and all-cause and cardiovascular mortality in AMI patients but also demonstrated that the combination of Lp(a) and high-sensitivity C-reactive protein (Hs-CRP) provided a better prediction of mortality risk (34). This predictive value of Lp(a) in combination with other biomarkers has also been validated in the general population, where Lp(a) was found to be independently associated with both all-cause and cardiovascular mortality. Additionally, when combined with fibrinogen, Lp(a) provided an even stronger predictive value for higher mortality risk compared to either marker alone (35). Furthermore, Kim et al. conducted a cohort study involving 275,430 Korean adults and found that high Lp(a) levels were associated with an increased risk of all-cause and cardiovascular mortality (36). After a follow-up period of 6.6 years, individuals with Lp(a) ≥ 50 mg/dL had a significantly higher risk of cardiovascular and all-cause mortality. Those with Lp(a) ≥ 100 mg/dL had a 2.45-fold increased risk of cardiovascular mortality compared to those with Lp(a) < 10 mg/dL. This association was independent of LDL-C and was only influenced by HDL-C levels, suggesting that Lp(a) is an independent risk factor for cardiovascular mortality in the Korean population. Beyond baseline Lp(a) levels, persistently high Lp(a) levels over long-term follow-up have also been strongly associated with mortality. For example, in a cohort study of 1,131 AMI patients, participants were categorized into four groups based on Lp(a) levels at admission and after one year: persistently low, increased, decreased, and persistently high Lp(a) levels (37). Over a median follow-up period of 50 months, the results indicated that, compared to the persistently low Lp(a) group, the persistently high Lp(a) group had significantly increased risks of major adverse cardiovascular and cerebrovascular events (MACCE), nonfatal stroke, unplanned revascularization, all-cause mortality, and cardiovascular mortality. This study demonstrated that persistently high Lp(a) levels in AMI patients were closely associated with an increased risk of MACCE, stroke, revascularization, and mortality.

Despite these compelling findings highlighting the strong association between Lp(a) and mortality risk, our study offers several distinct advantages compared to previous research. First, we utilized RCS analysis to examine the dose-response relationship between Lp(a) levels and mortality risk, confirming the linear nature of this association. This approach provides a clearer and more quantitative basis for the clinical application of Lp(a). Second, we selected 300 mg/L as the threshold for Lp(a) group division based on the upper reference limit commonly used in Chinese clinical laboratories (0-300 mg/L), which has also been widely adopted in domestic studies (38–40). In addition, we conducted a supplementary analysis using 500 mg/L, a widely recognized international cut-off value, and found that Lp(a) > 500 mg/L was significantly associated with increased cardiovascular mortality, further supporting the clinical relevance of this threshold. However, due to the retrospective nature of this study and incomplete clinical records, we were unable to accurately identify specific subgroups at extremely high cardiovascular risk, such as those with multivessel disease, peripheral artery disease, or familial hypercholesterolemia, and therefore could not perform further stratified analyses in these populations. Considering the greater vulnerability of these individuals to adverse outcomes, future prospective studies incorporating more detailed baseline data are warranted to clarify the prognostic significance of Lp(a) in this particularly high-risk group. In addition, current guidelines emphasize the need for intensified lipid-lowering therapy in extremely high-risk individuals (41–43). Although specific Lp(a)-targeted treatments are not yet widely available, such patients may benefit from aggressive LDL-C control through high-intensity statins, or ezetimibe. As Lp(a) contributes residual risk beyond traditional lipids, incorporating Lp(a) assessment into risk stratification could help identify those who may require more comprehensive therapeutic strategies. Third, our study was conducted in a cohort of Chinese ACS patients, filling a critical gap in the literature regarding the association between Lp(a) and all-cause and cardiovascular mortality in this population. This enhances the generalizability and relevance of our findings to Chinese patients. Fourth, we performed detailed subgroup and sensitivity analyses, further validating the robustness of our results and identifying specific high-risk populations, such as elderly males and individuals without comorbidities, who may be at an even greater risk of Lp(a)-related mortality. Moreover, due to the wide numerical range of Lp(a) concentrations, the risk increment associated with each 1 mg/dL increase is relatively small. Thus, although Lp(a) as a continuous variable shows statistically significant associations with mortality, the effect size per unit appears modest. To better reflect its clinical relevance, we additionally performed a standardized analysis, and the results indicated that a one standard deviation increase in Lp(a) was significantly associated with increased risks of both all-cause and cardiovascular mortality. This suggests that Lp(a) may have greater value in risk stratification when evaluated using standardized values or categorized by clinically meaningful thresholds to help identify individuals at higher risk. In summary, our study builds upon existing literature and further refines the clinical significance of Lp(a). These findings provide valuable insights for future precision medicine strategies aimed at optimizing risk assessment and management in ACS patients. And these results also support current international guideline recommendations advocating for at least one lifetime measurement of Lp(a), and suggest that such recommendations may also be applicable and beneficial in the Chinese ACS population (44). In addition, recent studies have further emphasized the importance of Lp(a) in CVD prevention and management (45, 46). For example, a review by Sosnowska et al. published in 2025 highlighted that Lp(a) is one of the current focal points in cardiovascular research (46). It not only contributes to the process of atherosclerosis but is also closely associated with aortic valve stenosis, inflammation, and thrombosis. The review emphasized that Lp(a) measurement should be incorporated into routine cardiovascular risk assessment and that the development of targeted Lp(a)-lowering therapies should be actively pursued. Future updates to local or regional clinical guidelines may consider integrating Lp(a) assessment as part of standard care for ACS patients.

Lp(a) may influence mortality risk in patients with ACS through multiple biological mechanisms. First, Lp(a) promotes atherosclerosis and thrombosis by facilitating atherosclerotic plaque formation through its cholesterol-rich LDL-like structure, while its Apo(a) component, which resembles plasminogen, competitively inhibits the fibrinolytic system, thereby enhancing thrombotic potential and increasing the risk of ACS recurrence (47–49). Second, Lp(a)-mediated inflammatory responses exacerbate cardiovascular damage by activating the monocyte-macrophage system, which stimulates the release of inflammatory cytokines such as interleukin-6 (IL-6) and tumor necrosis factor-alpha (TNF-α), thereby accelerating vascular inflammation and atherosclerosis progression (50, 51). Additionally, Lp(a) is closely associated with aortic valve and coronary artery calcification, microcirculatory dysfunction, and left ventricular hypertrophy, with elevated levels contributing to increased coronary plaque instability, impaired ventricular contractile function, reduced ejection capacity, and valvular regurgitation, ultimately heightening the susceptibility of ACS patients to sudden cardiac death and increasing long-term mortality risk (52–55). Overall, Lp(a) plays a crucial role in coagulation, inflammation, vascular injury, calcification, and myocardial hypertrophy, which may explain its strong association with poor prognosis in ACS patients.

Although this study provides important findings, certain limitations should be acknowledged. First, as a single-center retrospective study, there is a potential for selection bias, and the generalizability of the results needs further validation through large-scale, multicenter studies. Second, this study only measured baseline Lp(a) levels and did not assess the impact of temporal variations in Lp(a) on ACS prognosis. Although Lp(a) levels are relatively stable, they may still be influenced by acute inflammation or liver function changes in the short term. Future studies should consider dynamic monitoring of Lp(a) to more accurately evaluate its long-term effects. Third, despite adjusting for multiple confounders, residual confounding may still exist, as factors such as genetic polymorphisms and Lp(a) particle size were not included in the analysis and may have influenced the results. In addition, although comorbidities such as diabetes, hypertension, and CKD were included as covariates, they were defined only as binary variables. Detailed information on severity, duration, and control (such as blood pressure, blood glucose, and dynamic renal function) was not available. However, due to the retrospective nature of the study and reliance on historical electronic medical records, these data were largely missing. As a result, we were unable to incorporate more nuanced indicators of disease status, which may have limited the granularity of the multivariate models. Fourth, information on post-discharge treatment and medication adherence was not available due to limitations in retrospective data collection. Since long-term outcomes such as mortality are significantly affected by ongoing pharmacologic management—especially in ACS patients—this may introduce a potential source of bias. We recommend that future prospective studies include systematic follow-up of medication use to improve the accuracy and clinical relevance of outcome assessment. Fifth, although we performed comprehensive cardiovascular mortality analyses, the study did not incorporate competing risk models (such as accounting for non-cardiovascular death), which may be particularly relevant in older populations. Future research using competing risk approaches, such as Fine-Gray models, may help improve the accuracy and robustness of risk estimation. Lastly, given the lack of specific therapeutic interventions aimed at lowering Lp(a) levels, the findings of this study are primarily applicable to risk prediction rather than guiding treatment strategies. Further research is needed to explore whether lowering Lp(a) can improve clinical outcomes in ACS patients.

## 5 Conclusions

This study confirms a significant linear positive correlation between higher Lp(a) levels and all-cause and cardiovascular mortality in patients with ACS, with this association being more pronounced in specific subgroups, such as elderly males and those without comorbidities. The findings not only support Lp(a) as an independent prognostic predictor for ACS but also highlight the need for enhanced monitoring and management of patients with elevated Lp(a) levels in clinical practice. Future research should further explore the potential clinical benefits of Lp(a)-lowering therapies and integrate genetic studies to elucidate its biological mechanisms.

## Data Availability

The original contributions presented in the study are included in the article/supplementary material. Further inquiries can be directed to the corresponding author.
